# Supplementary material for: Study of risk perception consumption behavior of sports tourism in China
Source: PLoS One. 2023 Jul 18;18(7):e0288735. doi: 10.1371/journal.pone.0288735 (PMC10353816; doi:10.1371/journal.pone.0288735)
Supplement: Attachment — Submitted filename: Response to Reviewers.docx [file pone.0288735.s002.docx]

Response to Reviewers

Dear reviewers,

Thank you for your valuable suggestions on the revision of our paper. Your suggestions have guided the improvement of the paper and the thinking of future research.

According to the revision opinions, we have carefully revised the paper, and now we make the following answers to the review opinions:

**Reviewer #1:**

The study deals with a current topic, both the investigated area and the topic are relevant from the point of view of the research. The structure of the study is logical. It is recommended to present the correlations between the research questions and the applied methodology. The analysis of the performed tests is more descriptive, I recommend highlighting the correlations between the results. The obtained results are novel.

**Q1** present the correlations between the research questions and the applied methodology.

**Answer：**Add explanation in 4.1.2 line 209-218 as follows:

Multicollinearity means that the model estimation is distorted or difficult to estimate accurately due to the existence of precise correlation or high correlation between explanatory variables in the linear regression model. Generally speaking, due to the limitation of economic data, the model is improperly designed, resulting in a general correlation between explanatory variables in the design matrix.

This study investigates the relationship between factors such as gender, occupation, educational background, income and personality traits and perceived risk consumption behavior, so it is necessary to use multicollinearity analysis to prevent the model estimation distortion or difficulty to estimate accurately due to the existence of precise correlation or high correlation between dependent variables.

**Q2:**The analysis of the performed tests is more descriptive

**Answer：**The analysis of the performed tests is described in more detail, line 265, line 280-281, line 312-313, line334-335.

Reviewer #2:

**Q1:** The paper's title is quite complicated, hard to understand - it should be more clear.

**Answer**：Change the title to “Study of risk perception consumption behavior of sports tourism in China”

**Q2:**The abstract is more or less acceptable, however, it is very short and the first sentence is very long (5,5 rows...).

**Answer：**The abstract has been revised and the sentence has been adjusted.

**Q3:**The introduction is not acceptable, even not for a conference proceedings. It needs a vast extension, indicating the context and the research goals.

**Answer**: Background and objectives were added to the introduction based on comments, line 38-45, line 51, line 57-60.

**Q4:**The literature review isn't focused well, it is rather a theoretical framework which is fine but more international sources should be reviewed and analysed in a critical and analytical way.

**Answer:** The literature is reviewed and analyzed in a critical and analytical way.

**Q5**:It is not clear why the conclusions and suggestions are in one chapter. In fact, some points here are belonging rather to the conclusions chapter.
The conclusion is too short, it is not acceptable to make it in two sentences without real content and relevance.

**Answer:** 8.conclusion was rewritten

**Q6:**There is no real discussion in the paper and the description of the results is too technical, no real additions to the knowledge. The content is there but not articulated properly.

**Answer:** Added more discussion and result description in the paper.
