# Supplementary material for: Study of risk perception consumption behavior of sports tourism in China
Source: PLoS One. 2023 Jul 18;18(7):e0288735. doi: 10.1371/journal.pone.0288735 (PMC10353816; doi:10.1371/journal.pone.0288735)
Supplement: Attachment — Submitted filename: 6.23Response to the reviewer.docx [file pone.0288735.s003.docx]

Response to the reviewer:

1. Please remove your figures/ from within your manuscript file, leaving only the individual TIFF/EPS image files. These will be automatically included in the reviewer’s PDF.

Response：We have removed our figures from within the manuscript file, leaving only the individual TIFF/EPS image files.

2. Please ensure that you refer to Table 7 in your text as, if accepted, production will need this reference to link the reader to the Table.

Response：We have deleted table 7 in our text.
